# Differences in the Incidence of Hypotension and Hypertension between Sexes during Non-Cardiac Surgery: A Systematic Review and Meta-Analysis

**DOI:** 10.3390/jcm13030666

**Published:** 2024-01-24

**Authors:** Elke M. E. Bos, Johan T. M. Tol, Fabienne C. de Boer, Jimmy Schenk, Henning Hermanns, Susanne Eberl, Denise P. Veelo

**Affiliations:** 1 Department of Anaesthesiology, Amsterdam UMC, University of Amsterdam, Meibergdreef 9, 1105 AZ Amsterdam, The Netherlands; e.m.bos@amsterdamumc.nl (E.M.E.B.);; 2 Department of Intensive Care, Amsterdam UMC, University of Amsterdam, Meibergdreef 9, 1105 AZ Amsterdam, The Netherlands; 3 Department of Epidemiology and Data Science, Amsterdam UMC, University of Amsterdam, Meibergdreef 9, 1105 AZ Amsterdam, The Netherlands

**Keywords:** hemodynamics, intraoperative hypotension, perioperative care, sex differences, systematic review

## Abstract

*Background*: Major determinants of blood pressure (BP) include sex and age. In youth, females have lower BP than males, yet in advanced age, more pronounced BP increases result in higher average BPs in females over 65. This hypothesis-generating study explored whether age-related BP divergence impacts the incidence of sex-specific intraoperative hypotension (IOH) or hypertension. *Methods:* We systematically searched PubMed and Embase databases for studies reporting intraoperative BP in males and females in non-cardiac surgery. We analyzed between-sex differences in the incidence of IOH and intraoperative hypertension (primary endpoint). *Results*: Among 793 identified studies, 14 were included in this meta-analysis, comprising 1,110,636 patients (56% female). While sex was not associated with IOH overall (females: OR 1.10, 95%CI [0.98–1.23], *I*^2^ = 99%), a subset of studies with an average age ≥65 years showed increased exposure to IOH in females (OR 1.17, 95%CI [1.01–1.35], *I*^2^ = 94%). One study reported sex-specific differences in intraoperative hypertension, with a higher incidence in females (31% vs. 28%). *Conclusions*: While sex-specific reporting on intraoperative BP was limited, IOH did not differ between sexes. However, an exploratory subgroup analysis offers the hypothesis that females of advanced age may face an increased risk of IOH, warranting further investigation.

## 1. Introduction

Since the beginning of the 1990s, biological sex has been increasingly recognized as an important factor in cardiovascular (patho)physiology with numerous significant differences between females and males in cardiovascular disease [1]. In 2022, the European Society of Cardiology published a scientific statement on sex-specific differences in arterial hypertension, highlighting possible sex-specific physiological and pathophysiological mechanisms [2]. Suggested mechanisms range from differences in gene expression, sex hormones, and incidence of autoimmune disorders to sex-specific physiological events such as menarche, pregnancy, and menopause [2]. Young and middle-aged females tend to have lower blood pressure (BP) compared to males; however, due to a steeper rise after midlife, females ≥65 years of age show higher BP, i.e., systolic blood pressure (SBP) and mean arterial pressure (MAP), than males [2,3]. While monitoring and maintenance of intraoperative BP is a core element of anesthesia practice, it is currently unknown whether these sex-related differences in physiology also result in clinically relevant differences in intraoperative hemodynamic stability.

Many publications are available on the topic of intraoperative BP in general and intraoperative hypotension specifically. Most are retrospective cohort studies reporting various thresholds of arterial pressure associated with adverse outcomes such as cardiac injury, kidney injury, and mortality, without any regard for sex-specific risks [4,5,6,7,8,9]. Several studies that analyzed sex as a potential risk factor of hypotension in heterogeneous populations found conflicting results, with some finding that female sex is associated with increased incidence of hypotension [10,11,12], others finding that male sex is associated with intraoperative hypotension (IOH) [13,14], or finding no sex-related differences [15]. We performed this hypothesis-generating systematic review and meta-analysis to investigate the potential presence of sex-specific differences in exposure to hypo- or hypertension during anesthesia for non-cardiac surgery.

## 2. Methods

This systematic review was performed according to the methodologic guidelines outlined in the Cochrane handbook for systematic reviews [16] and in adherence with the 2020 PRISMA statement [17]. The study protocol was drafted and submitted to the PROSPERO registry (ID: CRD42023394177) prior to the systematic search of the literature.

### 2.1. Search Strategy

A systematic search was conducted in the PubMed and Embase (Ovid) databases on 24 February 2023 in cooperation with a clinical librarian. Articles were identified using synonyms and medical subject headings from a selection of different terms including those of male, female, sex factors, blood pressure, hypertension, hypotension, and perioperative care. The full search strategy can be found in Supplementary S1. Titles, abstracts, and full text were independently screened for eligibility by three reviewers (E.M.E.B., J.T., and F.C.d.B.) using the online review program Rayyan [18]. When no consensus could be reached, a fourth author (HH) was consulted to form a final decision.

### 2.2. Eligibility

Studies were included when relevant information on intraoperative BP (e.g., absolute values for systolic/diastolic/mean arterial pressure, relative values for arterial pressure, i.e., incidence of intraoperative hyper- or hypotension based on predetermined definitions by the reporting authors, vasopressor, or fluid administration) was reported for both adult females and males undergoing non-cardiac surgery under general or neuraxial anesthesia. Studies were ineligible when the language was something other than Dutch, German, or English or when full texts were unavailable. Single case reports, review articles, and literature studies were excluded as well as studies reporting on patients with congenital heart disease or on circulatory support such as extracorporeal membrane oxygenation or left ventricular assist device. If multiple records were reported for the same patient cohort, only the report with the largest number of patients was included to prevent patient overlap.

### 2.3. Outcome

The primary aim was to explore possible differences in the incidence of intraoperative hypo- or hypertension between male and female sexes during non-cardiac surgery. Secondary outcomes were sex-specific differences in fluid and vasopressor administration.

### 2.4. Data Extraction

Data extraction was independently performed by three reviewers (E.M.E.B, J.T., and F.C.d.B.). Data on the study and patient characteristics, definitions of arterial pressure and measurement methods, and the incidence of hypo- or hypertension in both males and females were extracted. When available, adjusted odds ratios based on multivariate analysis were used for the final analysis to ensure the use of the most accurate effect size. The average (mean or median) age of the cohort was extracted from each study or calculated manually when the number of patients was reported per age range. The three reviewers subsequently performed quality assessments of the included studies using the Newcastle–Ottawa scale (NOS) for observational research reports [19]. Studies with NOS lower than five out of a maximum of nine points were excluded from the final meta-analysis to reduce the risk of bias from low-quality studies. See Supplemental S2 in the Supplemental Data for the Newcastle–Ottawa quality assessment scale.

### 2.5. Statistical Analysis

A meta-analysis was performed using the extracted study data. Data were compiled and analyzed in a random-effects model using R version 4.2.1 (R foundation, Vienna, Austria) using the meta and metafor packages [20]. If one study reported multiple thresholds of IOH, e.g., both MAP < 65 mmHg and <55 mmHg, only the most severe threshold (<55 mmHg) of IOH was included in our primary meta-analysis to ensure the inclusion of the clinically most impactful threshold as increasing severity of IOH is associated with adverse clinical outcomes [9,21]. The difference in exposure to IOH or intraoperative hypertension was reported as an odds ratio (OR) with a 95% confidence interval (CI). The Mantel–Haenszel method was used to pool studies and statistical heterogeneity was quantified using the I^2^ statistic [16]. Since we expected considerable heterogeneity in the definition of outcome measures such as hypotension, we pre-specified sensitivity and subgroup analyses. A funnel plot was constructed and both were visually inspected and tested for publication bias, using Egger’s test [22].

### 2.6. Sensitivity Analyses

We performed two sensitivity analyses.

1. Using the least severe threshold of IOH when multiple thresholds for IOH were reported in one study, as relying exclusively on the most severe threshold of IOH could lead to the omission of numerous cases of milder hypotension;
2. Using only studies that were classified as generalizable based on the NOS scale (question S1, representativeness of the exposed cohort) to assess the possible influence of the generalizability of the study population on the overall meta-analysis.

### 2.7. Subgroup Analyses

We performed two subgroup analyses.

1. Analyzing the studies with a mean or median age of ≥65 years and those with a mean or median age of <65 years, since BP differences between the sexes vary through age [3] and an increase in BP is observed in post-menopausal women [23]. Even though substantial overlap in age may be present between the relatively older and younger subgroups, we accepted this limitation due to biological plausibility of a potential difference in BP combined with the exploratory nature of the present study;
2. Analyzing the studies only reporting absolute thresholds (e.g., MAP < 65 mmHg for IOH), only reporting relative thresholds (e.g., >30% reduction in MAP for IOH), and studies reporting a combination of absolute and relative thresholds, with the rationale that the baseline difference in blood pressure between sexes is accounted for in relative thresholds, thus obscuring potential sex-differences.

## 3. Results

**Figure 1:**
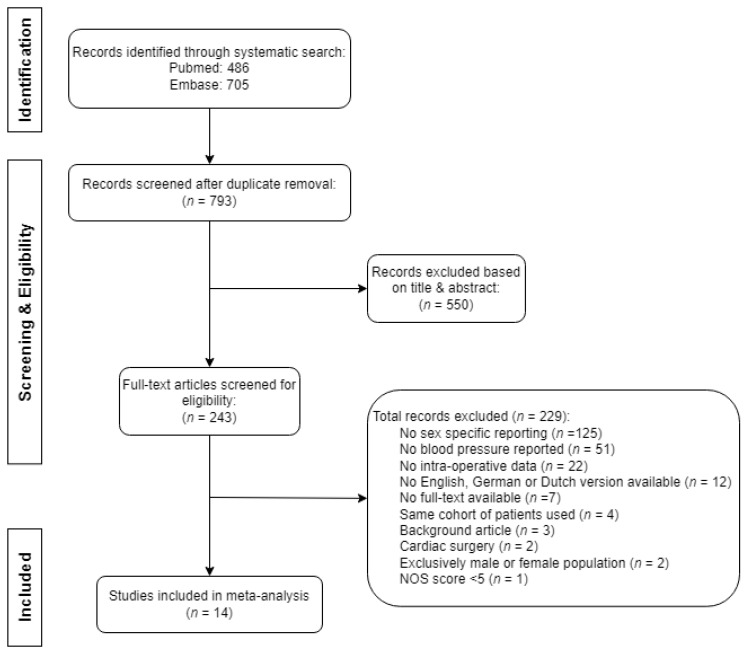
Flowchart of the search and selection of records for review.

Of the 1191 retrieved publications, we selected 243 full-text articles for assessment of eligibility, after which we included 14 records in this review. The selection of records is described in Figure 1. The main reasons for exclusion based on title and/or abstract comprised the surgical population (e.g., cardiothoracic surgery), presence of congenital heart disease, use of circulatory support, or the timing of blood pressure measurements (pre- or postoperative only). Further clarification for the exclusion of articles based on full-text analysis is explained in Figure 1. The primary reason for exclusion, as determined through full-text analysis, was the absence of sex-specific reporting, accounting for 55% of articles (125 out of 229). One record was excluded due to low quality, defined as a NOS score of four [24]. No additional articles were found by screening the corresponding reference lists.

A total of 1,110,636 patients, of which 620,661 (56%) females, were included in the analyses. Of the 14 articles, 1 reported sex-based differences in the incidence of both IOH and intraoperative hypertension, the remaining 13 articles reported differences in IOH exclusively, 3 articles reported on post-induction hypotension (PIH), and 10 articles reported on IOH from induction to emergence of anesthesia, thus including PIH as well. The incidence of hypotension during non-cardiac surgery was observed to be 87,180 (26%) in males and 130,382 (28%) in females. Two studies reported the association between sex and IOH, without reporting the incidence of IOH for either sex separately [12,25].

The only study describing intraoperative hypertension analyzed BP during non-cardiac surgery in 16,079 patients, of which 8316 (52%) were females. The incidence of intraoperative hypertension, defined as a systolic BP > 160 mmHg, was 2197 (28%) in males and 2557 (31%) in females.

Actual blood pressure values (i.e., in mmHg) were not reported for females and males separately in any of the included studies. The characteristics of the included reports are shown in Table 1. NOS score for all included studies are shown in Table 2.

### 3.1. Meta-Analysis

**Figure 2:**
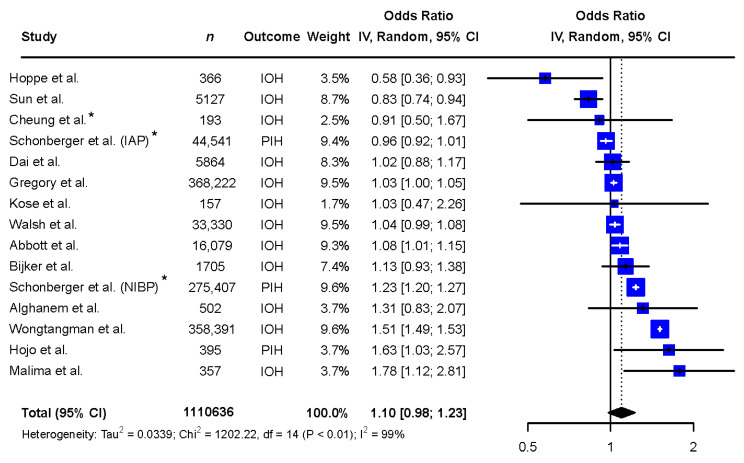
Meta-analysis of the included records describing the difference in exposure to intraoperative hypotension between the sexes. Abbreviations: *n*: number, CI: confidence interval, df: degrees of freedom, IAP: invasive arterial pressure, IOH: intraoperative hypotension, NIBP: non-invasive blood pressure, PIH: post-induction hypotension. * Adjusted odds ratio based on multivariate analysis [6,9,10,11,12,13,14,15,25,26,27,28,29,30].

**Figure 3:**
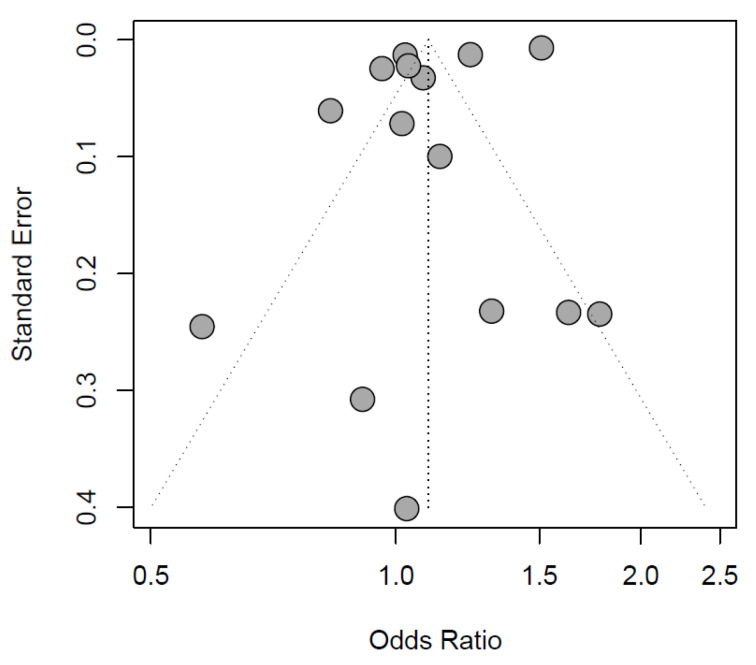
Funnel plot of the included records.

The exploratory meta-analysis showed no significant difference in exposure to IOH between sexes (females: OR 1.10 [0.98 to 1.23], reference males, Figure 2). Study designs and populations varied greatly. Accordingly, statistical heterogeneity was high (I^2^ = 99%, *p* < 0.01). Egger’s test of the funnel plot showed no indication of publication bias (*p*-value = 0.1047, Figure 3).

### 3.2. Sensitivity Analyses

The two sensitivity analyses, i.e., using the least severe threshold for IOH (females: OR 1.12 [1.01 to 1.25], *I^2^* = 98%, reference males) and representative study cohorts (females: OR 1.08 [0.94 to 1.24], I^2^ = 99%, reference males) did not yield different results to the main meta-analysis and did not reduce heterogeneity, see Figures S1 and S2 in Supplemental S3.

### 3.3. Subgroup Analyses

**Figure 4:**
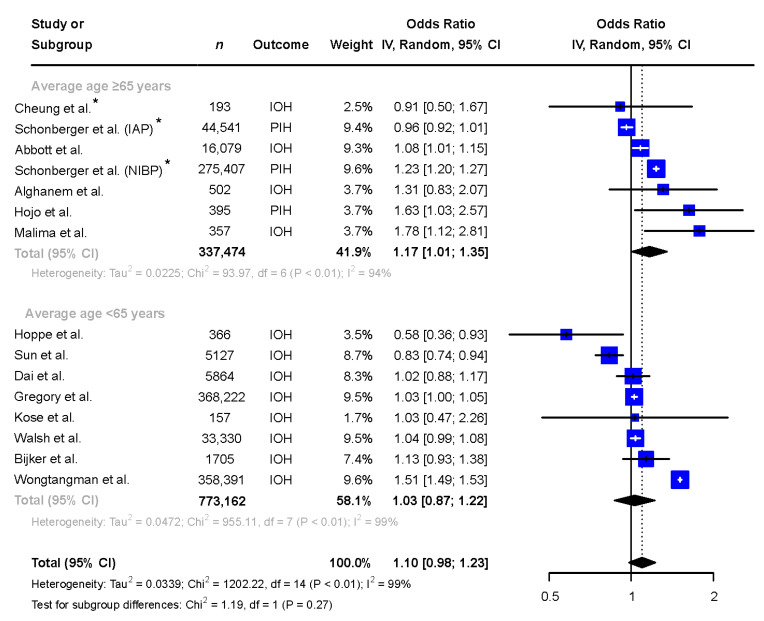
Subgroup analysis comparing the exposure to intraoperative hypotension in males and females in reports with mean/median age of the patient population ≥65 and <65 years. * Adjusted odds ratio based on multivariate analysis. Abbreviations: *n*: number, CI: confidence interval, df: degrees of freedom, IAP: invasive arterial pressure, IOH: intraoperative hypotension, NIBP: non-invasive blood pressure, PIH: post-induction hypotension [6,9,10,11,12,13,14,15,25,26,27,28,29,30].

In studies with a mean or median age ≥65 years (*n* = 337,474, 53% female), female patients were more likely to be exposed to hypotension when compared to male patients and the heterogeneity remained high (OR 1.17 [1.01 to 1.35], I^2^ = 94%), see Figure 4. In studies with a mean or median age below 65 years of age (*n* = 773,162, 53% female), no difference between sexes in exposure to IOH was seen (OR 1.03 [0.87 to 1.22], I^2^ = 99%), see Figure 4.

The second subgroup analysis, focusing on absolute, relative, or combined thresholds, showed no difference in exposure to IOH between sexes, see Figure S3 in Supplemental S3 (absolute thresholds: females: OR 1.11 [0.98 to 1.26], I^2^ = 99%, relative thresholds: females: OR 0.91 [0.53 to 1.56], I^2^ = 67%, or combined thresholds: females: OR 1.17 [0.81 to 1.69], I^2^ = 63%, reference males). The subgroup that defined IOH based on absolute thresholds included the vast majority of patients (*n* = 1,087,129), while the remaining subgroups consisted of relatively small total numbers of patients (relative thresholds: *n* = 1025 and combined thresholds: *n* = 6414).

### 3.4. Secondary Outcomes

We were unable to extract sex-specific vasopressor dose or cumulative fluid administration from any of the included studies. As such, the planned secondary analyses could not be performed.

## 4. Discussion

This systematic review and meta-analysis aimed to examine possible sex-related differences in intraoperative blood pressure during non-cardiac surgery. Our overall hypothesis-generating meta-analysis showed no difference in exposure to intraoperative hypotension between the sexes. Based on an exploratory subgroup analysis, focusing on selected studies involving an overall older patient population (i.e., mean/median age ≥65 years), we propose the hypothesis that older females may experience increased exposure to IOH during general or neuraxial anesthesia for non-cardiac surgery. However, due to the inherent risks of significant heterogeneity, we want to emphasize that definite conclusions cannot be made and this finding should only be used as a rationale for future investigations. Regarding hypertension, only one of the included studies reported sex-specific exposure to intraoperative hypertension, precluding further analyses.

The most important conclusion of this systematic review is the overall paucity of evidence concerning biological sex and intraoperative blood pressure during non-cardiac surgery. Through our systematic search, we identified 14 studies reporting intraoperative blood pressure stratified by sex; however, in the current body of literature, we could not identify any records reporting absolute intraoperative BP values nor could we find any studies reporting on the extent of IOH (depth and duration), dose of vasopressors, or volume of fluids stratified by sex. Moreover, 10 out of 14 studies included in our analysis reported the prevalence of pre-existing (pre-operative) hypertension; however, none of them categorized these data by sex. The use and effectiveness of pre-operative antihypertensive medication presented in the included studies was not sex-stratified either. Consequently, we were unable to incorporate these aspects into our analyses. Given that the premise of the study was grounded in population-level disparities in arterial blood pressure between sexes, the inclusion of such information would have provided valuable insights. Similarly, considering the potential impact of surgery duration on hypotension incidence, it is noteworthy that only 6 out of 14 studies reported the duration of surgery, with none of these studies presenting data stratified by sex. Nonetheless, there is no indication to suggest significant variation in procedure duration between sexes within the study populations. Despite the likelihood that numerous researchers focusing on perioperative hemodynamics presumably have access to data on sex and blood pressure, we opted not to reach out to authors if pertinent data were unavailable in the published manuscript. This decision was made a priori, considering the potential substantial number of eligible studies and in recognition of the hypothesis-generating nature of the current study. The lack of sex-specific reporting found in the current study corresponds to the findings of a 2016 study evaluating surgical research projects between 2011–2012, which found that 38% reported detailed sex findings, 33% analyzed sex-based findings, and 23% discussed sex-based results [31]. In light of recent publications focusing on sex-related differences in cardiovascular health [2,32] and increased incidence of long-term major adverse cardiac and cerebrovascular events after cardiac surgery in female patients [33,34,35], this lack of reporting is remarkable.

The findings of our exploratory subgroup analysis, indicating a potential increase in exposure to intraoperative hypotension (IOH) among elderly females undergoing general or neuraxial anesthesia for non-cardiac surgical procedures, underscore the importance of investigating sex-specific cardiovascular physiology, which transcends beyond the intraoperative setting. Research on sex-related distinctions in cardiovascular physiology within the non-anesthetized population indicates that females aged 65 and above, typically postmenopausal [3,36,37], generally exhibit higher blood pressures than males. Studies have proposed that males undergoing antihypertensive therapy tend to achieve better BP control than females and that these sex-based disparities increase with age, with the greatest disparities occurring in individuals over 75 years of age [38]. The etiology of such variations remains unknown and could stem from biological factors, suboptimal treatment modalities (physicians inertia, patient non-adherence, and inappropriate drug selection), or other comorbidities [2,39]. Furthermore, despite a delayed onset of vascular disease in women relative to men by 10 to 20 years, recent data suggest that the risk of cardiovascular complications and organ damage emerges at lower BP levels [2,40,41] and progresses faster in females compared to males [3], questioning current practice of using the same BP threshold for the identification of hypertension for both sexes [2].

Arterial hypertension (with or without medication) and age are reported risk factors associated with IOH [15,42]. However, the association between a preoperative history of hypertension and increased intraoperative cardiovascular lability is ambiguous [42,43]. Moreover, various factors, including hormonal, chromosomal, and gene expression variance, as well as socioeconomic and environmental influences, contribute to the physiological disparities observed between females and males [44]. These multifaceted elements may play a role in explaining the increased susceptibility of older females to IOH. Females are predisposed to coronary microvascular dysfunction, accelerated arterial stiffening, and increased pulse pressure, which may contribute to an elevated risk of heart failure with preserved ejection fraction and an increased incidence of left ventricular diastolic dysfunction (LVDD), particularly in the presence of vascular risk factors such as hypertension [45]. LVDD, in turn, reduces left ventricular end-diastolic volume, thereby impacting stroke volume and directly influencing cardiac output [46].

Moreover, controversy surrounding the importance of intraoperative blood pressure in itself has persisted for decades [47]. Ensuring adequate perfusion and oxygenation of body tissues [21,48], involves a complex interplay of multiple factors regulating both blood pressure and flow. The data derived from this hypothesis-generating study provide substantial grounds for further exploration into sex-specific differences in the extent of deviation from baseline blood pressure, duration of IOH, or intraoperative hypertension, as well as the type, dose, and effectiveness of treatment of BP abnormalities. Female vasculature, even when adjusted for body surface area, is relatively smaller and stiffer compared to males [3], which may influence the impact of vasopressor treatment on end-organ blood flow. Arterial stiffness is less modifiable by antihypertensive therapy in females than in males [2]. The implications of arterial stiffness on the effects of vasoactive agents during general or neuraxial anesthesia remain unknown. If we assume that females over 65 within this study received comparable doses of vasoactive agents and/or fluid boluses as their male counterparts, the higher exposure to IOH in older females might refer to refractory hypotension, which is associated with an increase in perioperative morbidity [21,29,48,49]. Nevertheless, the above-mentioned factors are speculative considerations for the potential etiology of increased intraoperative cardiovascular instability in older females. This study does not allow for a comprehensive examination of this topic and the etiology remains of interest to future research.

While significant progress has been made in our understanding of sex differences in BP abnormalities over the past decades, a notable portion of this knowledge awaits clinical adoption [2]. Furthermore, the 2022 European Society of Cardiology (ESC) Guidelines on cardiovascular assessment and management of patients undergoing non-cardiac surgery currently lack any reference to clinical integration [50]. Despite recognizing the pivotal role of age in preoperative diagnostics and monitoring, specifically at 65 and above, the guidelines refrain from recommending differentiated approaches based on sex. This underscores the necessity for further research to unravel the nuanced interactions between age, sex, and cardiovascular physiology. The overarching objective is to refine preventive and management strategies for both females and males undergoing non-cardiac surgery.

### 4.1. Limitations

Major limitations of our meta-analysis are the substantial heterogeneity and the paucity of data, posing significant interpretative challenges. We chose to present our exploratory meta-analysis, despite these limitations, because there is biological plausibility for a difference in exposure to either intraoperative hypo- or hypertension between males and females. This is based on population-level research that demonstrates distinct BP trajectories between the sexes in the awake non-anesthetized population [2,3]. Furthermore, the included studies differed with respect to the chosen definitions of IOH. Nine different definitions for hypotension were found, comprising absolute thresholds, relative thresholds, and a combination of both. Even though we performed sensitivity analyses to assess the influence of these different IOH definitions, the in-group differences regarding IOH definition were considerable, which we were unable to correct. Moreover, as described in the methods section, the results of the subgroup analyses (i.e., mean/median <65 years and mean/median age ≥65 years) should be interpreted with caution, since subgroup heterogeneity remained high and some overlap between the two populations was inevitable due to the available data in the underlying records. Finally, although not indicated by the funnel plot, underreporting and publication bias may have influenced our results.

While acknowledging these caveats, the present analyses can improve our understanding of differences between sexes in exposure to intraoperative BP abnormalities. Future research is needed to explore the etiology, impact, and management of intraoperative hypo- and hypertension considering both age and sex as critical factors in optimizing intraoperative hemodynamic stability during non-cardiac surgery.

### 4.2. Conclusions

This systematic review highlights the scarcity and heterogeneity of sex-specific reporting on intraoperative BP, posing significant challenges to the interpretation of the results. We found no evidence to suggest either males or females are more exposed to intraoperative hypo- or hypertension overall. However, based on an exploratory subgroup analysis, we tentatively propose the hypothesis that older females (i.e., mean/median age ≥65 years) may experience increased exposure to IOH during general or neuraxial anesthesia for non-cardiac surgery.

## Figures and Tables

**Table 1:** Characteristics of the included reports.

Author	*n*	Female (%)	Age	Surgery type	Surgery Duration (in Min)	Anesthesia Type (%)	Other Selection Criteria	History of Arterial Hypertension	Method BP Measurement	Outcome	Hypo/Hypertension Threshold	Minimum Duration	Incidence Men	Incidence Women	Incidence Overall
Abbott, 2018 [26]	16,079	8316 (51.7%)	65 (11.9)	Noncardiac	NS	Regional and/or GA	≥1 day hospital LOS	8171 (50.8%)	NIBP	IOH	SBP < 100 mmHg	NS	4702 (61%)	5189 (62%)	9891 (62%)
										IOHT	SBP > 160 mmHg	NS	2197 (28%)	2557 (31%)	4754 (30%)
Alghanem, 2020 [27]	502	237 (47.2%)	71.7 (14.2)	Unilateral femur fracture	NS	Spinal (74.3%) or GA (25.7%)	None	291 (58.0%)	NIBP or IAP	IOH	≥30% reduction in SBP	≥10 min	43 (16%)	48 (20%)	91 (18%)
Bijker, 2009 [6]	1705	825 (48.4%)	52 (15.8)	General or vascular surgery	112 (IQR 73–163)	Neuraxial (11.8%) GA (71.9%) Combined (16.3%)	None	380 (22.3%)	NIBP or IAP	IOH	SBP < 80 mmHg	≥1 min	324 (37%)	328 (40%)	652 (38%)
Cheung, 2015 [25]	193	128 (66.3%)	67.6 (11.3)	Noncardiac	NS	Neuraxial (23.3%) GA (69.4%) Combined (7.3%)	On loop diuretic on regular basis before surgery. Loop diuretic or placebo on day of surgery.	NS	NIBP or IAP	IOH	SBP < 90 mmHg OR > 35% decrease in MAP OR administration of vasopressors	≥5 min *	NS	NS	107 (55%)
Dai, 2020 [15]	5864	3103 (52.9%)	47.4 *	Noncardiac	NS	Neuraxial (6.9%) GA (92.2%) IA (4.7%) GA + NB (2.9%) GA + IA (0.2%)	None	855 (14.5%)	NIBP or IAP	IOH	SBP < 90 mmHg OR > 20% decrease in MAP	≥10 min	435 (16%)	496 (16%)	931 (16%)
Gregory, 2021 [9]	368,222	226,694 (62%)	60.1 *	Noncardiac, non-cesarian	30 (IQR 16–60)	Not specified	≥1 day hospital LOS	230,482 (63%)	NIBP or IAP	IOH	MAP < 75 mmHg	≥1 measurement	51,725 (37%)	94,018 (41%)	14,5743 (40%)
										IOH	MAP < 65 mmHg	≥1 measurement	25,874 (18%)	45,064 (20%)	70,938 (19%)
										IOH	MAP < 55 mmHg	≥1 measurement	10,402 (7%)	17,071 (8%)	27,473 (7%)
Hojo, 2022 [11]	395	184 (47%)	70 [61–78]	Oral/maxillofacial	NA	GA	Hypertensive patients, on medication	395 (100%)	NIBP or IAP	PIH	MAP < 55 mmHg	≥1 min	87 (41%)	101 (55%)	188 (48%)
Hoppe, 2022 [13]	366	195 (53%)	52 [47–57]	Elective noncardiac	51 (IQR 30–84)	GA	ASA class I or II, no DM, no CHF, no CKD	87 (24.0%)	NIBP or IAP	PIH	≥30% reduction in MAP compared to preoperative nighttime MAP	NS	84 (49%)	76 (39%)	160 (44%)
										IOH	≥30% reduction in MAP compared to preoperative nighttime MAP	NS	71 (42%)	54 (28%)	125 (34%)
Kose, 2012 [28]	157	75 (47.8%)	45.3 (10.8)	Elective noncardiac	127.8 (SD 35.7)	GA	ASA class I or II, no betablockers/calcium channel blockers	29 (18.5%)	Continuous non invasive BP	IOH	>30% reduction in MAP	NS	16 (20%)	15 (20%)	31 (20%)
Malima, 2019 [10]	357	212 (59.4%)	67 (8.3)	Lower limb surgery	NS	Spinal	None	NS	NIBP	IOH	≥25% reduction in SBP or SBP < 100 mmHg	NS	68 (47%)	132 (62%)	200 (56%)
Schonberger, 2022 [12]	275,470	142,672 (51.8%)	72.8 (6.3)	Non-minor surgery	192.1 (SD 110.3)	GA	GA using propofol	NS	NIBP	PIH	MAP < 55 mmHg	NS	NS	NS	57,009 (20.7%)
	44,541	19,477 (43.7%)	73.7 (6.6)	Non-minor surgery	302.3 (SD 139.3)	GA	GA using propofol	NS	IAP	PIH	MAP < 55 mmHg	NS	NS	NS	15,589 (35.0%)
Sun, 2015 [14]	5127	2708 (53%)	61.3 (14.2)	Noncardiac, non-urologic	<2 h 509 (31.9%) 2–5 h 874 (54.7%) >5 h 214 (13.4%)	GA	≥1 day hospital LOS, invasive BP monitoring and etCO2 available	2437 (47.5%)	IAP	IOH	MAP < 65 mmHg	Various durations reported	2281 (94%)	2530 (93%)	4811 (94%)
										IOH	MAP < 60 mmHg	Various durations reported	2097 (87%)	2276 (84%)	4373 (85%)
										IOH	MAP < 55 mmHg	Various durations reported	1716 (71%)	1814 (67%)	3530 (69%)
Walsh, 2013 [29]	33,330	16,836 (50.5%)	55.8 (15.5)	Noncardiac, non-urologic	NS	GA	≥1 day hospital LOS, pre- and post-operative creatinine	NS	NIBP or IAP	IOH	MAP < 55 mmHg	≥1 measurement	7024 (43%)	7317 (43%)	14,341 (43%)
Wongtangman, 2021 [30]	358,391	198,915 (55.5%)	54 (16.6)	Noncardiac	NS	GA	None	144,880 (40.4%)	NIBP or IAP	IOH	MAP < 55 mmHg	NS	62,292 (39%)	97,817 (49%)	160,109 (45%)

Data presented as number (%), mean (standard deviation) or median [25th–75th percentiles]. Abbreviations:, *n*: number, BP: blood pressure, GA: general anesthesia, IA: intrathecal anesthesia, NS: not specified, NB: neuraxial block, LOS: length of stay, ASA: American Society of Anesthesiologists physical status, DM: diabetes mellitus, CHF: congestive heart failure, CKD: chronic kidney disease, IAP: intra-arterial pressure, IOH: intraoperative hypotension, IOHT: intraoperative hypertension, NIBP: non-invasive blood pressure, NS: not specified, PIH: post-induction hypotension, SBP: systolic blood pressure, MAP: mean arterial pressure, IQR: interquartile rage, SD: standard deviation. * Mean age calculated post hoc using age ranges provided in articles.

**Table 2:** Newcastle–Ottawa scale * of the included reports.

Author	Year	Study Design	S1	S2	S3	S4	C	O1	O2	O3	Total NOS
Abbott [26]	2018	Multicenter prospective cohort study	1	1	1	0	2	1	1	1	8
Alghanem [27]	2020	Single center retrospective cohort study	0	1	1	1	0	1	1	1	6
Bijker [6]	2009	Single center retrospective cohort study	1	1	1	1	0	1	1	1	7
Cheung [25]	2015	Single center prospective cohort study	0	1	1	1	2	0	1	0	6
Dai [15]	2020	Cohort study using data of multicenter RCT	1	1	1	1	2	1	1	0	8
Gregory [9]	2021	Single center retrospective cohort study	0	1	1	1	0	1	1	1	6
Hojo [11]	2022	Multicenter retrospective cohort study	0	1	1	0	2	1	0	1	6
Hoppe [13]	2022	Prospective observational stugy	1	1	1	1	2	1	1	1	9
Kose [28]	2012	Prospective observational study	0	1	1	1	0	1	1	0	5
Malima [10]	2019	Single center retrospective cohort study	1	1	1	1	2	1	1	1	9
Schonberger	2022	Multicenter retrospective cohort study	1	1	1	1	2	1	1	1	9
Sun [14]	2015	Single center prospective cohort study	1	1	1	1	2	1	1	1	9
Walsh [29]	2013	Single center retrospective cohort study	1	1	1	0	0	1	1	0	5
Wongtangman [30]	2021	Single center prospective cohort study	1	1	1	1	0	1	1	1	7

* see Supplemental S2 for full instrument.

## Supplementary Materials

The following supporting information can be downloaded at https://www.mdpi.com/article/10.3390/jcm13030666/s1.

## Abbreviations

aOR	Adjusted odds ratio
BP	Blood pressure
DBP	Diastolic blood pressure
IOH	Intraoperative hypotension
MAP	Mean arterial pressure
NOS	Newcastle–Ottawa scale
OR	Odds ratio
PIH	Post induction hypotension
SBP	Systolic blood pressure
