# Supplementary material for: Differences in the Incidence of Hypotension and Hypertension between Sexes during Non-Cardiac Surgery: A Systematic Review and Meta-Analysis
Source: J Clin Med. 2024 Jan 24;13(3):666. doi: 10.3390/jcm13030666 (PMC10856734; doi:10.3390/jcm13030666)
Supplement: Supplementary file 1 [file jcm-13-00666-s001.zip › jcm-2782176-supplementary.pdf]

Supplemental data

Supplemental S1

Search strategy. Search was performed on February 24th, 2023. The comprehensive literature search string was prepared with the advice of a clinical librarian.

PUBMED

("Female"[MeSH] OR "Male"[MeSH] OR "Sex Factors"[MeSH] OR female*[tiab] OR woman[tiab] OR women[tiab] OR men[tiab] OR male[tiab] OR males[tiab] OR sex[tiab] OR sexes[tiab] OR sex difference*[tiab] OR sex factor*[tiab] OR sex-specific*[tiab] OR sex categor*[tiab] OR sex related[tiab] OR gender difference*[tiab]) AND ("Blood Pressure"[Mesh] OR "Hypertension"[Mesh] OR "Hypotension"[Mesh] OR blood pressur*[tiab] OR mean arterial pressur*[tiab] OR MAP[tiab] OR MABP[tiab] OR systolic pressur*[tiab] OR diastolic pressur*[tiab] OR systolic blood pressur*[tiab] OR systolic BP[tiab] OR SBP[tiab] OR diasystolic blood pressur*[tiab] OR diastolic BP[tiab] OR hypertens*[tiab] OR hypotens*[tiab]) AND ("Perioperative Period"[Mesh] OR "Perioperative Care"[Mesh] OR "Monitoring, Intraoperative"[Mesh] OR perioperat*[tiab] OR peri-operat*[tiab] OR preoperat*[tiab] OR pre-operat*[tiab] OR intraoperat*[tiab] OR intra-operat*[tiab] OR postoperat*[tiab] OR post-operat*[tiab] OR during surg*[tiab] OR during operat*[tiab] OR undergo*[tiab]) AND (non-cardiac*[tiab] OR noncardiac*[tiab]) NOT ("Animals"[Mesh] NOT "Humans"[Mesh]) NOT ("Child"[Mesh] NOT "Adult"[Mesh]) NOT (pediatric*[ti] OR paediatric*[ti] OR child*[ti] OR cardiac surg*[ti] OR animal*[ti] OR cat[ti] OR cats[ti])

EMBASE (OVID):

#	Searches	Results
1	female/ or male/ or *sex factor/ or *sex difference/ or *sex/	15345053
2	(female* or woman or women or men or male or males or sex or sexes or sex difference* or sex factor* or sex-specific* or sex categor* or sex related or gender difference*).ti,ab,kf.	5640732
3	1 or 2	16069387
4	*blood pressure/ or mean arterial pressure/ or *diastolic blood pressure/ or exp *systolic blood pressure/ or hypertension/ or hypotension/	1006511
5	(blood pressur* or mean arterial pressur* or MAP or MABP or systolic pressur* or diastolic pressur* or systolic blood pressur* or systolic BP or SBP or diasystolic blood pressur* or diastolic BP or hypertens* or hypotens*).ti,ab,kf.	1485330
6	4 or 5	1816834
7	perioperative period/ or perioperative monitoring/ or intraoperative monitoring/	65675
8	(perioperat* or peri-operat* or preoperat* or pre-operat* or intraoperat* or intra-operat* or postoperat* or post-operat* or during surg* or during operat* or undergo*).ti,ab,kf.	2499219

9	7 or 8	2509798
10	(non-cardiac* or noncardiac*).ti,ab,kf.	22347
11	3 and 6 and 9 and 10	1223
12	animal/ not human/	1584018
13	11 not 12	1223
14	child/ not adult/	1613658
15	13 not 14	1196
16	(pediatric* or paediatric* or child* or cardiac surg* or animal* or cat or cats).ti.	1673158
17	15 not 16	955
18	limit 17 to conference abstract status	250
19	17 not 18	705

Supplemental S2

Newcastle-Ottawa quality assessment scale

CASE CONTROL STUDIES

Note: A study can be awarded a maximum of one star for each numbered item within the Selection and Exposure categories. A maximum of two stars can be given for Comparability.

Selection

1) Is the case definition adequate?

  a) yes, with independent validation

  b) yes, eg record linkage or based on self reports

  c) no description

2) Representativeness of the cases

  a) consecutive or obviously representative series of cases

  b) potential for selection biases or not stated

3) Selection of Controls

  a) community controls

  b) hospital controls

  c) no description

4) Definition of Controls

  a) no history of disease (endpoint)

  b) no description of source

Comparability

1) Comparability of cases and controls on the basis of the design or analysis

  a) study controls for _______________ (Select the most important factor.)

  b) study controls for any additional factor (This criteria could be modified to indicate specific control for a second important factor.)

Exposure

1) Ascertainment of exposure

  a) secure record (eg surgical records)

b) structured interview where blind to case/control status

c) interview not blinded to case/control status

d) written self report or medical record only

e) no description

2) <u>Same method of ascertainment for cases and controls</u>

a) yes

b) no

3) <u>Non-Response rate</u>

a) same rate for both groups

b) non respondents described

c) rate different and no designation

NEWCASTLE - OTTAWA QUALITY ASSESSMENT SCALE

COHORT STUDIES

<u>Note</u>: A study can be awarded a maximum of one star for each numbered item within the Selection and Outcome categories. A maximum of two stars can be given for Comparability

Selection

1) <u>Representativeness of the exposed cohort</u>

a) truly representative of the average _______________ (describe) in the community

b) somewhat representative of the average ______________ in the community

c) selected group of users eg nurses, volunteers

d) no description of the derivation of the cohort

2) <u>Selection of the non exposed cohort</u>

a) drawn from the same community as the exposed cohort

b) drawn from a different source

c) no description of the derivation of the non exposed cohort

3) <u>Ascertainment of exposure</u>

a) secure record (eg surgical records)

b) structured interview

c) written self report

d) no description

4) <u>Demonstration that outcome of interest was not present at start of study</u>

a) yes

b) no

Comparability

1) <u>Comparability of cohorts on the basis of the design or analysis</u>

a) study controls for _____________ (select the most important factor)

b) study controls for any additional factor (This criteria could be modified to indicate specific control for a second important factor.)

Outcome

1) <u>Assessment of outcome</u>

a) independent blind assessment

b) record linkage

c) self report

d) no description

2) Was follow-up long enough for outcomes to occur

a) yes (select an adequate follow up period for outcome of interest)

b) no

3) Adequacy of follow up of cohorts

a) complete follow up - all subjects accounted for

b) subjects lost to follow up unlikely to introduce bias - small number lost - > ____ % (select an adequate %) follow up, or description provided of those lost)

c) follow up rate < ____% (select an adequate %) and no description of those lost

d) no statement

Supplemental S3

Study	*n*	Outcome	Weight	Odds Ratio IV, Random, 95% CI
Hoppe et al.	366	IOH	3.3%	0.58 [0.36; 0.93]
Sun et al.	5127	IOH	6.9%	0.86 [0.68; 1.08]
Cheung et al. *	193	IOH	2.4%	0.91 [0.50; 1.67]
Schonberger et al. (IAP) *	44,541	PIH	9.8%	0.96 [0.92; 1.01]
Dai et al.	5864	IOH	8.5%	1.02 [0.88; 1.17]
Kose et al.	157	IOH	1.6%	1.03 [0.47; 2.26]
Walsh et al.	33,330	IOH	9.8%	1.04 [0.99; 1.08]
Abbott et al.	16,079	IOH	9.7%	1.08 [1.01; 1.15]
Bijker et al.	1705	IOH	7.5%	1.13 [0.93; 1.38]
Gregory et al.	368,222	IOH	10.0%	1.23 [1.21; 1.25]
Schonberger et al. (NIBP) *	275,407	PIH	10.0%	1.23 [1.20; 1.27]
Alghanem et al.	502	IOH	3.6%	1.31 [0.83; 2.07]
Wongtangman et al.	358,391	IOH	10.0%	1.51 [1.49; 1.53]
Hojo et al.	395	PIH	3.5%	1.63 [1.03; 2.57]
Malima et al.	357	IOH	3.5%	1.78 [1.12; 2.81]
**Total (95% CI)**	**1,110,636**		**100.0%**	**1.12 [1.01; 1.25]**

Heterogeneity: $Tau^2 = 0.0297$; $Chi^2 = 884.60$, df = 14 ($P < 0.01$); $I^2 = 98\%$

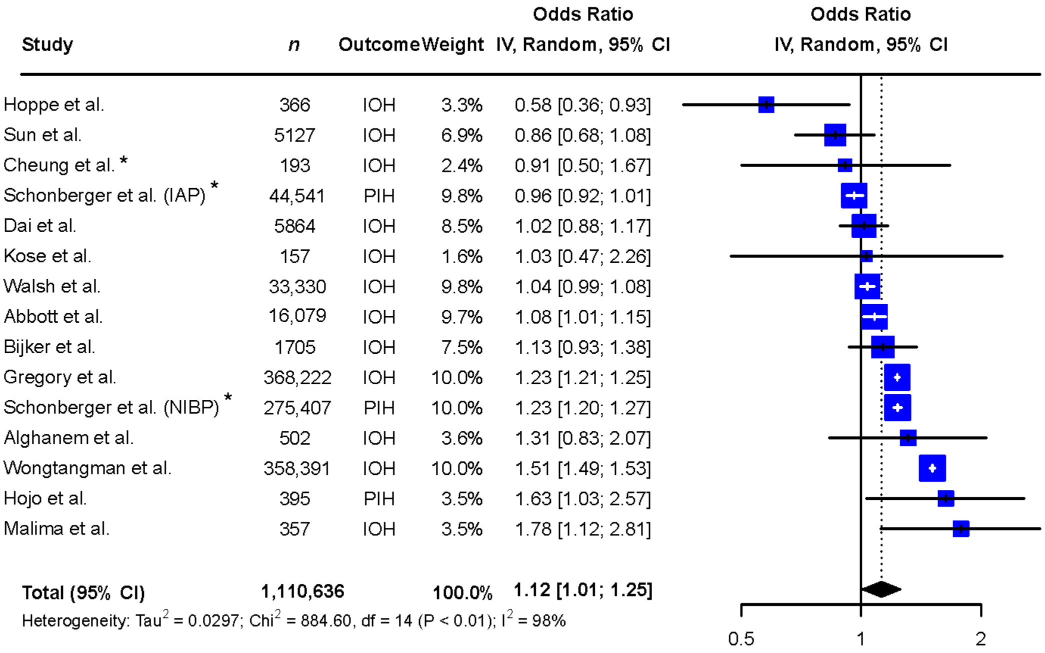

Figure S1. Sensitivity analysis: Least severe definition of intraoperative hypotension [6,9–15,25–28,30].
Abbreviations: *n*: number, CI: confidence interval, df: degrees of freedom, IAP: invasive arterial pressure, IOH: intraoperative hypotension, NIBP: non-invasive blood pressure, PIH: post-induction hypotension
* Adjusted odds ratio based on multivariate analysis

Study	N	Outcome	Weight	Odds Ratio IV, Random, 95% CI
Hoppe et al.	366	IOH	4.9%	0.58 [0.36; 0.93]
Sun et al.	5127	IOH	10.9%	0.83 [0.74; 0.94]
Schonberger et al. (IAP)*	44,541	PIH	11.7%	0.96 [0.92; 1.01]
Dai et al.	5864	IOH	10.6%	1.02 [0.88; 1.17]
Walsh et al.	33,330	IOH	11.7%	1.04 [0.99; 1.08]
Abbott et al.	16,079	IOH	11.6%	1.08 [1.01; 1.15]
Bijker et al.	1705	IOH	9.6%	1.13 [0.93; 1.38]
Schonberger et al. (NIBP)*	275,407	PIH	11.8%	1.23 [1.20; 1.27]
Wongtangman et al.	358,391	IOH	11.9%	1.51 [1.49; 1.53]
Malima et al.	357	IOH	5.2%	1.78 [1.12; 2.81]
**Total (95% CI)**	**741,167**		**100.0%**	**1.08 [0.94; 1.24]**

Heterogeneity: $Tau^2 = 0.0427$; $Chi^2 = 792.89$, df = 9 ($P < 0.01$); $I^2 = 99\%$

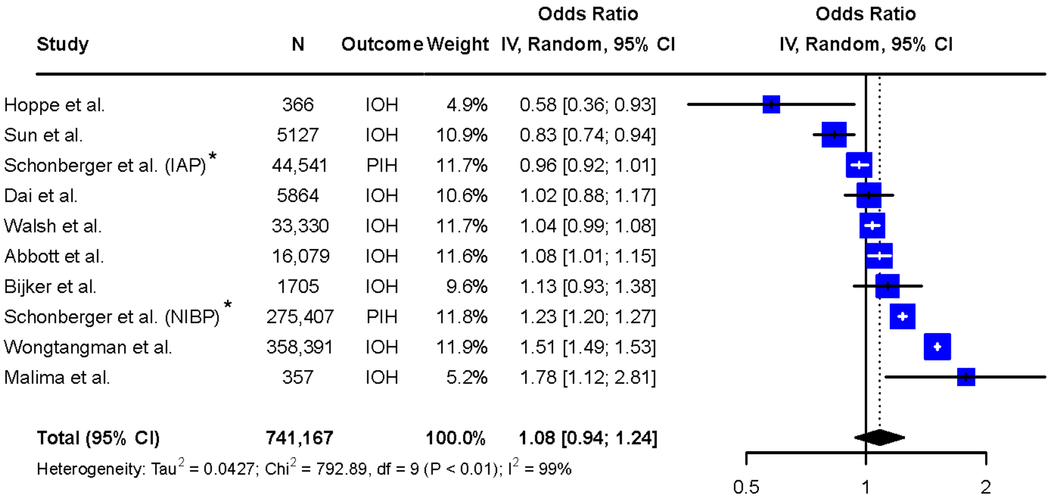

Figure S2. Sensitivity analysis: Representative study cohorts [6,10,12–14,26,29,30].
Abbreviations: *n*: number, CI: confidence interval, df: degrees of freedom, IAP: invasive arterial pressure, IOH: intraoperative hypotension, NIBP: non-invasive blood pressure, PIH: post-induction hypotension
* Adjusted odds ratio based on multivariate analysis

Study or Subgroup	*n*	Outcome	Weight	Odds Ratio IV, Random, 95% CI
Absolute Threshold				
Sun et al.	5127	IOH	8.7%	0.83 [0.74; 0.94]
Schonberger et al. (IAP) *	44,541	PIH	9.4%	0.96 [0.92; 1.01]
Gregory et al.	368,222	IOH	9.5%	1.03 [1.00; 1.05]
Walsh et al.	33,330	IOH	9.5%	1.04 [0.99; 1.08]
Abbott et al.	16,079	IOH	9.3%	1.08 [1.01; 1.15]
Bijker et al.	1705	IOH	7.4%	1.13 [0.93; 1.38]
Schonberger et al. (NIBP) *	275,407	PIH	9.6%	1.23 [1.20; 1.27]
Wongtangman et al.	358,391	IOH	9.6%	1.51 [1.49; 1.53]
Hojo et al.	395	PIH	3.7%	1.63 [1.03; 2.57]
**Total (95% CI)**	**1,087,129**		**76.6%**	**1.11 [0.98; 1.26]**
Heterogeneity: $Tau^2 = 0.0319$; $Chi^2 = 1175.57$, df = 8 ($P < 0.01$); $I^2 = 99\%$				
Relative Threshold				
Hoppe et al.	366	IOH	3.5%	0.58 [0.36; 0.93]
Kose et al.	157	IOH	1.7%	1.03 [0.47; 2.26]
Alghanem et al.	502	IOH	3.7%	1.31 [0.83; 2.07]
**Total (95% CI)**	**1025**		**8.8%**	**0.91 [0.53; 1.56]**
Heterogeneity: $Tau^2 = 0.1406$; $Chi^2 = 5.99$, df = 2 ($P = 0.05$); $I^2 = 67\%$				
Combined Threshold				
Cheung et al. *	193	IOH	2.5%	0.91 [0.50; 1.67]
Dai et al.	5864	IOH	8.3%	1.02 [0.88; 1.17]
Malima et al.	357	IOH	3.7%	1.78 [1.12; 2.81]
**Total (95% CI)**	**6414**		**14.5%**	**1.17 [0.81; 1.69]**
Heterogeneity: $Tau^2 = 0.0687$; $Chi^2 = 5.45$, df = 2 ($P = 0.07$); $I^2 = 63\%$				
**Total (95% CI)**	**1,110,636**		**100.0%**	**1.10 [0.98; 1.23]**
Heterogeneity: $Tau^2 = 0.0339$; $Chi^2 = 1202.22$, df = 14 ($P < 0.01$); $I^2 = 99\%$				
Test for subgroup differences: $Chi^2 = 0.58$, df = 2 ($P = 0.75$)				

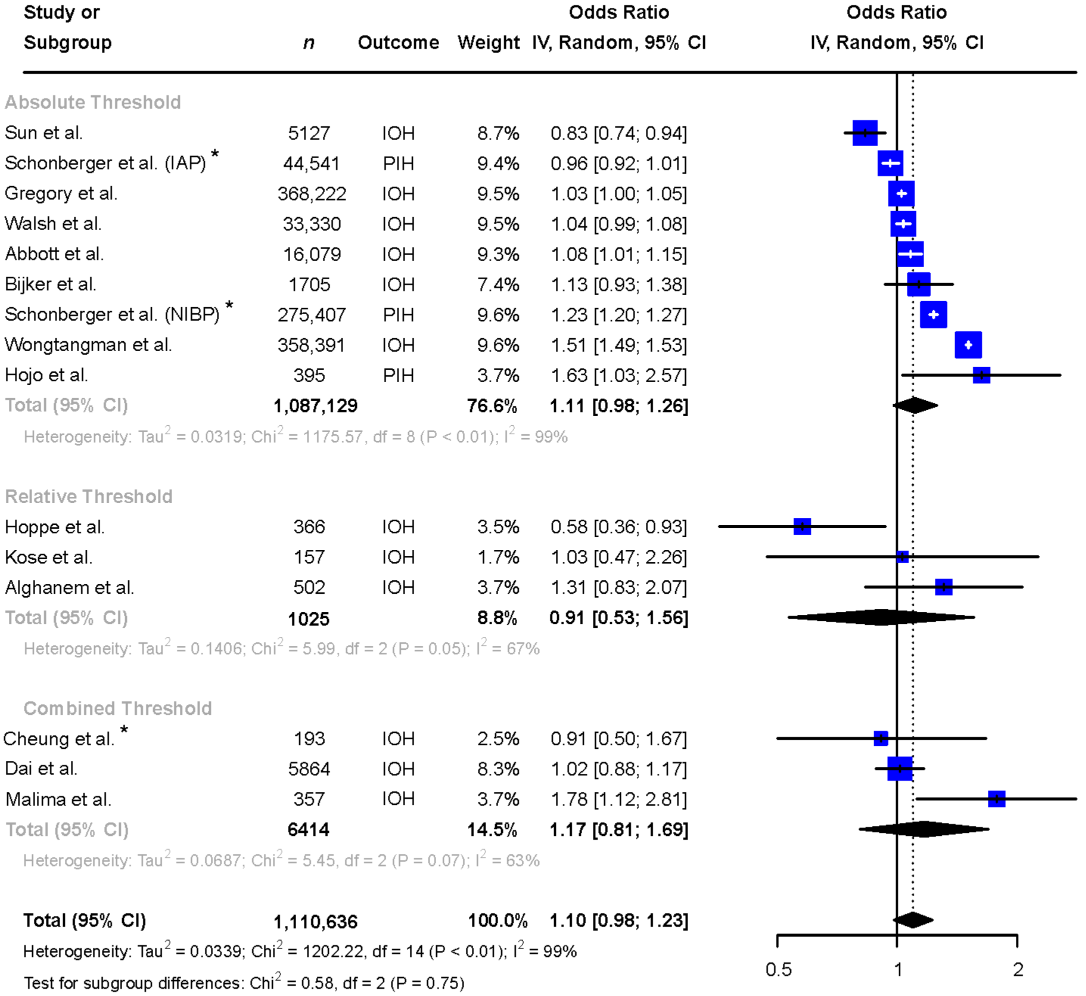

Figure S3. Subgroup analysis: absolute versus relative versus combined thresholds for intraoperative hypotension [6,9–15,25–30].

Abbreviations: *n*: number, CI: confidence interval, df: degrees of freedom, IAP: invasive arterial pressure, IOH: intraoperative hypotension, NIBP: non-invasive blood pressure, PIH: post-induction hypotension

* Adjusted odds ratio based on multivariate analysis
